# The performance of ChatGPT-4.0o in medical imaging evaluation: a cross-sectional study

**DOI:** 10.3352/jeehp.2024.21.29

**Published:** 2024-10-31

**Authors:** Elio Stefan Arruzza, Carla Marie Evangelista, Minh Chau

**Affiliations:** 1 UniSA Allied Health & Human Performance, University of South Australia, Adelaide, SA, Australia; 2 Jones Radiology, Eastwood, SA, Australia; 3 School of Dentistry and Medical Sciences, Charles Sturt University, Wagga Wagga, NSW, Australia; Hallym University, Korea

**Keywords:** Artificial intelligence, Radiography, Diagnostic imaging, Radiology

## Abstract

This study investigated the performance of ChatGPT-4.0o in evaluating the quality of positioning in radiographic images. Thirty radiographs depicting a variety of knee, elbow, ankle, hand, pelvis, and shoulder projections were produced using anthropomorphic phantoms and uploaded to ChatGPT-4.0o. The model was prompted to provide a solution to identify any positioning errors with justification and offer improvements. A panel of radiographers assessed the solutions for radiographic quality based on established positioning criteria, with a grading scale of 1–5. In only 20% of projections, ChatGPT-4.0o correctly recognized all errors with justifications and offered correct suggestions for improvement. The most commonly occurring score was 3 (9 cases, 30%), wherein the model recognized at least 1 specific error and provided a correct improvement. The mean score was 2.9. Overall, low accuracy was demonstrated, with most projections receiving only partially correct solutions. The findings reinforce the importance of robust radiography education and clinical experience.

## Graphical abstract

[Fig f2-jeehp-21-29]

## Introduction

### Background/rationale

Artificial intelligence (AI) is leading to significant transformations in healthcare and radiology [1]. One of these innovations is ChatGPT by Open AI, a language model that can generate human-like text responses based on prompts by a human user.

In radiology, previous literature has demonstrated the accuracy of ChatGPT in interpreting medical images for pathological findings. A recent systematic review evaluated the reported performance of ChatGPT within radiology and found that 19 of 24 studies recorded a median accuracy of 70.5%, while the remaining 5 studies had a median agreement of 83.6% between ChatGPT outcomes and the radiologists’ decisions [2]. Nevertheless, cited concerns in the radiology literature about using ChatGPT included biased responses, limited originality, and the potential for inaccurate information leading to misinformation [2]. While these studies highlight ChatGPT’s advances in interpreting medical images, they solely focus on diagnostic interpretation, rather than critiquing the technical quality of the images. Globally, the scope of practice of a medical imaging technologist, or diagnostic radiographer specifically involves a requirement to “critically analyze clinical images for technical quality and suggest improvement if required” [3], and “apply quality criteria to assure image quality and evaluate medical images” [4].

A key factor influencing the need to repeat a radiographic exposure is how well the positioning of the patient satisfies radiographic positioning guidelines. Proper positioning ensures that relevant anatomical structures are visible, in the correct orientation and with minimal distortion, allowing radiologists to accurately interpret the radiograph. The need to repeat an image when it does not meet diagnostic criteria has both health and financial implications [5]. For example, repeatably exposing the patient to radiation poses a risk of cancer development, even at low doses [6]. Furthermore, the increased time and cost associated with further exposures, as well as the consumption of materials and wear-and-tear on equipment [7], can affect the effectiveness of a radiology department. Exploring ways to improve radiographer positioning and reduce repeat rates, will therefore help to minimize exposure and have benefits for department financially.

### Objectives

In radiography education specifically, ChatGPT has been touted to improve digital literacy and graduate outcomes of students while streamlining the preparation process for educators [8]. However, a previous iteration (ChatGPT-3.0) introduced errors and fabricated information, as well as exhibiting a lack of depth of insight and appropriateness for professional communication education [9]. Furthermore, these studies did not have access to the new iteration’s image upload ability, which can be used to assess how the model evaluates image-based data. This study therefore aimed to address this gap by investigating the accuracy of ChatGPT-4.0o in evaluating the quality of radiographs, focusing particularly on positioning. By examining its performance in this context, this project sought to provide valuable insights pertaining to the potential of generative AI in radiography education and practice.

## Methods

### Ethics statement

Ethics approval was not required since it is not a study of human or animal subjects.

### Study design/setting

This study employed a cross-sectional design to evaluate the accuracy of ChatGPT-4.0o in critiquing radiographs for diagnostic quality, particularly for optimal positioning. Radiographs demonstrating a variety of knee, elbow, ankle, hand, shoulder, and pelvic projections were produced using various anthropomorphic phantoms. The phantoms were purposely positioned to include a range and spectrum of positioning errors, including radiographs that were optimally positioned.

Each radiograph was reviewed by a panel of 3 radiographers (E.A., C.E., & M.C.) with 15 years of cumulative clinical experience to determine the radiographic positioning error(s) and describe the most suitable improvement(s) to enhance the projection. Furthermore, each radiographer was directly involved with the positioning of the phantoms, and hence could determine instructions for improvement through visualization of the phantom in the X-ray room. The panel assessed the images for radiographic quality based on predefined criteria, following established standardized positioning criteria [10].

**Fig. 1.**
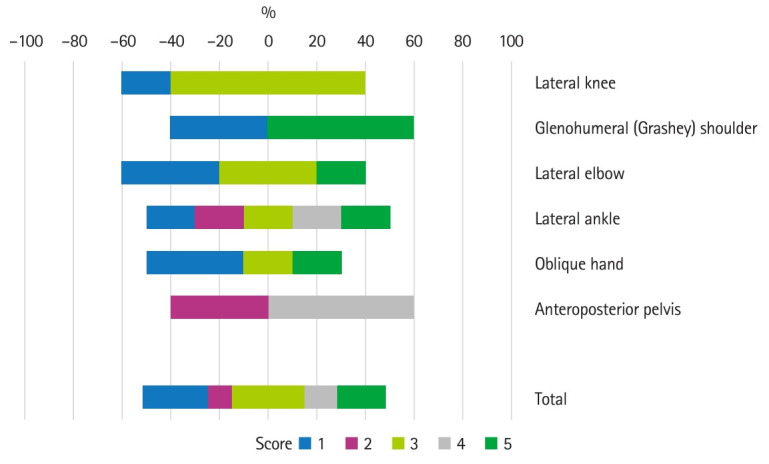
Graphical representation of scores.

Using the “image upload” tool, each radiograph was uploaded into ChatGPT, and was prompt was entered. The prompt comprised the following tasks: (1) to identify the positioning error(s), (2) to explain the error using specific and relevant anatomical terminology, and (3) to provide a suitable radiographic method to enhance the positioning. The model generated a text response summarizing its evaluation of the positioning. A sample prompt has been provided in Fig. 1. The initial prompts and radiographs used can be found in Supplement 1.

### Variables/data sources/measurement

The texts generated by ChatGPT-4.0o were then assessed against the solution provided by the expert panel. A scoring system was employed to quantify ChatGPT’s performance. Each critique was rated on a scale from 1 to 5, with 1 indicating a complete failure to identify any error(s) (or incorrectly identify an optimal image as having error[s]), and 5 indicating a comprehensive and accurate critique. The evaluation of the ChatGPT’s responses were also collectively evaluated by the expert panel. solutions conducted by the entire panel. The specific scoring rubric is seen in Table 1.

### Study size

Given the nature of this preliminary investigation, no study size was estimated. The methods utilized have not been used prior to this study.

### Statistical methods

Descriptive statistics were used to analyze the data using IBM SPSS ver. 28.0 (IBM Corp.), and the results were tabulated and graphed appropriately in Microsoft Excel 2021 (Microsoft Corp.). Furthermore, a narrative discussion regarding the insights gained by investigators whilst undertaking the experiment was written.

## Results

Thirty radiographs were sourced and entered into ChatGPT-4.0o, consisting of: lateral knee (n=5), lateral ankle (n=5), anteroposterior pelvis (n=5), oblique hand (n=5), glenohumeral (Grashey) shoulder (n=5) and lateral elbow (n=5) projections. ChatGPT-4.0o correctly recognized all errors with justifications and offered correct improvements (i.e., exact match accuracy) for 6 projections (20%). In 8 cases (26.67%), ChatGPT-4.0o ether did not recognize any specific error(s) present or incorrectly identified an optimal image as having error(s) (i.e., scored 0). The accuracy of the model in recognizing at least 1 specific error and provided a correct improvement (i.e., a score of 3, 4, 5) was 63.33%. The most commonly occurring score was 3 (9 cases or 30%), whereby the model recognized at least 1 specific error and provided a correct improvement. The mean score was 2.9 (1.47) and the median score was 3. Considering only the mean score, the highest performing projection was the glenohumeral shoulder, whilst the worst performing projections were the lateral knee and oblique hand. A summary of the critiques for each projection type is demonstrated in Table 2 and Fig. 1.

## Discussion

### Key results

This preliminary investigation explored the performance of ChatGPT-4.0o in evaluating the quality of radiographs, focusing specifically on radiographic positioning. This is the first study to use any iteration of ChatGPT for this task. With consideration of the criticality for radiographers to comprehensively evaluate a radiographer with total accuracy, ChatGPT-4.0o demonstrated low accuracy. However, the model demonstrated greater accuracy in identifying at least 1 error and suggesting an associated improvement, with most projections resulting in partially correct solutions by the model.

Medical imaging technologists are professionals with advanced critical thinking skills [11], developed through theoretical learning and hands-on experience in real-life clinical situations. In the era of ChatGPT’s current iteration, the findings of this study reinforce the importance of robust radiography education and clinical experience, in training competent radiographers with critical-thinking abilities. Nevertheless, given the emphasis the field of radiography places on continuous professional development [3,4], it is equally important to be aware of and promote the integration of tools and techniques that may improve healthcare practice.

### Interpretation/comparison with previous studies

The magnitude and nature of errors produced by ChatGPT-4.0o in this experiment are somewhat concerning given the increase in awareness and use of generative AI by health and medical students [12,13]. For many of the projections, incorrect information was often paired with a lack of specificity relating to justification of the noted error and the associated improvement. Relating to the elbow projections, for instance, ChatGPT often only critiqued the changes needed to lift or depress the wrist/forearm, whilst providing little or no insight into how the height of the shoulder/humerus influences the appearance of elbow structures. For the knee projections particularly, the model often only critiqued the degree of condylar rotation; it provided minimal to no information relating to errors involving the inferior aspect of the condyles, which are influenced by abduction/adduction of the leg, or some degree of caudal/cephalad tube angulation. On some occasions, the output would make the user aware of the criteria that classify images as optimal but did not necessarily apply this theoretical knowledge to the image provided. For the radiographer, a deficiency in understanding specific corrective technique is arguably as significant as erroneous approaches to improve positioning, as ultimately, both issues may result in incorrect decision-making and higher repeat rates.

Although the literature may be scarce in radiography, ChatGPT-4.0 has shown major improvement in radiological performance compared to previous iterations [14]. It is probable that AI models will only continue to improve, as models are reinforced with radiology-specific imaging and language data [15]. Given the parallels between radiological and radiographic language and data, it is anticipated these enhancements will also have implications for radiography-related use of generative AI.

### Limitations

This study was not without limitations. Firstly, a small magnitude of prompt engineering (i.e., initial prompts and providing additional prompts to guide algorithm output) was utilized. It is therefore possible that the quality of the output could be enhanced by more suitable and extensive prompting. Nevertheless, the short and simplistic nature of the prompts reflected the probable use of the program by students and radiographers in educational and clinical settings. Secondly, the images critiqued did not present the entire range of image projections and anatomical variance.

### Suggestions

Expanding the dataset to include more radiographs from a variety of human sources, including those with differing anatomical features and alternative presentations of technical quality, would allow more comprehensive testing of ChatGPT. Positioning is just 1 indicator of radiographic quality, and other indicators such as exposure and anatomical coverage could also be evaluated.

### Conclusion

In its current iteration, ChatGPT-4.0o demonstrated low accuracy in evaluating the positioning of radiographs. The number of errors produced in this study underscores the need for further refinement of the model; nevertheless, its potential as an educational or assistive tool within radiography is evident. The ability of generative AI to offer rapid feedback may be advantageous; however, the core abilities of critical thinking and problem-solving remain essential for radiographers.

## Figures and Tables

**Figure f2-jeehp-21-29:**
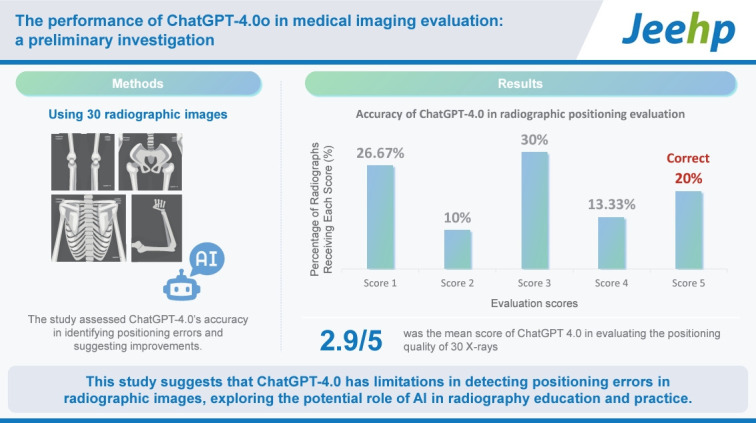


**Table 1.** Grading system used to score responses

Score				
1	2	3	4	5
Did not recognize any specific error(s) present or incorrectly identified an optimal image as having error(s)	Recognized at least 1 specific error, but did not provide correct explanation(s) or improvements	Recognized at least 1 specific error and provided a correct improvement	Recognized all error(s), but provided 1 or more incorrect explanation(s) or improvements	Recognized all errors, thoroughly explained with justification, and offered correct improvement(s)

**Table 2.** Frequency of scores for each projection

	Score					
	1	2	3	4	5	Mean±SD
Lateral knee	1	0	4	0	0	2.6
Glenohumeral shoulder (Grashey)	2	0	0	0	3	3.4
Lateral elbow	2	0	2	0	1	2.6
Lateral ankle	1	1	1	1	1	3
Oblique hand	2	0	2	0	1	2.6
Anteroposterior pelvis	0	2	0	3	0	3.2
Total	8 (26.67)	3 (10.00)	9 (30.00)	4 (13.33)	6 (20.00)	2.9±1.47

Values are presented as number, number (%), or mean±SD. SD, standard deviation.

## Supplementary materials

Supplementary files are available from Harvard Dataverse: https://doi.org/10.7910/DVN/QQ8PKO

Supplement 1. Dataset of radiographs.

Supplement 2. Audio recording of the abstract.

## References

[1] Khalifa M, Albadawy M (2024). AI in diagnostic imaging: revolutionising accuracy and efficiency. Comput Methods Programs Biomed Update.

[2] Keshavarz P, Bagherieh S, Nabipoorashrafi SA, Chalian H, Rahsepar AA, Kim GHJ, Hassani C, Raman SS, Bedayat A (2024). ChatGPT in radiology: a systematic review of performance, pitfalls, and future perspectives. Diagn Interv Imaging.

[3] Health and Care Professions Council (2023). Standards of proficiency: radiographers [Internet]. https://www.hcpc-uk.org/globalassets/resources/standards/standards-of-proficiency---radiographers.pdf.

[4] Australian Health Practitioner Regulation Agency, Medical Radiation Practice Board (2020). Professional capabilities for medical radiation practice [Internet]. https://gcgglobalhealthcare.com/wp-content/uploads/2020/11/Professional-Capabilities-for-Medical-Radiation-Practice.pdf.

[5] Al-Malki MA, Abulfaraj WH, Bhuiyan SI, Kinsara AA (2003). A study on radiographic repeat rate data of several hospitals in Jeddah. Radiat Prot Dosimetry.

[6] Hasaneen M, AlHameli N, AlMinhali A, Alshehhi S, Salih S, Alomaim MM (2023). Assessment of image rejection in digital radiography. J Med Life.

[7] Almalki AA, Abdul Manaf R, Hanafiah Juni M, Kadir Shahar H, Noor NM, Gabbad A (2017). Educational module intervention for radiographers to reduce repetition rate of routine digital chest radiography in Makkah region of Saudi Arabia tertiary hospitals: protocol of a quasi-experimental study. JMIR Res Protoc.

[8] Amedu C, Ohene-Botwe B (2024). Harnessing the benefits of ChatGPT for radiography education: a discussion paper. Radiography (Lond).

[9] Currie G, Singh C, Nelson T, Nabasenja C, Al-Hayek Y, Spuur K (2023). ChatGPT in medical imaging higher education. Radiography (Lond).

[10] Lampignano JP, Kendrick LE (2018). Bontrager’s textbook of radiographic positioning and related anatomy.

[11] Pieterse T, Temane A, Downing C (2023). A model to facilitate critical thinking of radiography students. J Med Radiat Sci.

[12] Yigit S, Berse S, Dirgar E, Gulhan Guner S (2024). Views of health sciences undergraduates on ChatGPT, an artificial intelligence-powered language model: a qualitative study. Innov Educ Teach Int.

[13] Zhang JS, Yoon C, Williams DK, Pinkas A (2024). Exploring the usage of ChatGPT among medical students in the United States. J Med Educ Curric Dev.

[14] Shieh A, Tran B, He G, Kumar M, Freed JA, Majety P (2024). Assessing ChatGPT 4.0's test performance and clinical diagnostic accuracy on USMLE STEP 2 CK and clinical case reports. Sci Rep.

[15] Lourenco AP, Slanetz PJ, Baird GL (2023). Rise of ChatGPT: it may be time to reassess how we teach and test radiology residents. Radiology.

