# Supplementary material for: The performance of ChatGPT-4.0o in medical imaging evaluation: a cross-sectional study
Source: J Educ Eval Health Prof. 2024 Oct 31;21:29. doi: 10.3352/jeehp.2024.21.29 (PMC11586623; doi:10.3352/jeehp.2024.21.29)
Supplement: Supplementary file 1 — Supplement 1. Dataset of radiographs. [file jeehp-21-29-suppl1.docx]

**Supplement 1.** Dataset of radiographs

**Lateral ankle**

Prompt: This is a lateral ankle projection. In terms of radiographic positioning, please determine the errors, if any, in the radiograph? If there are errors, please determine the nature of the error(s) and provide instructions for a radiographer to improve the image.

| 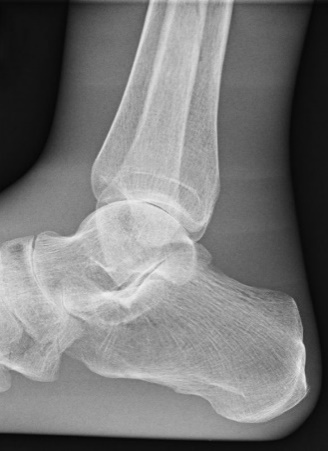 | 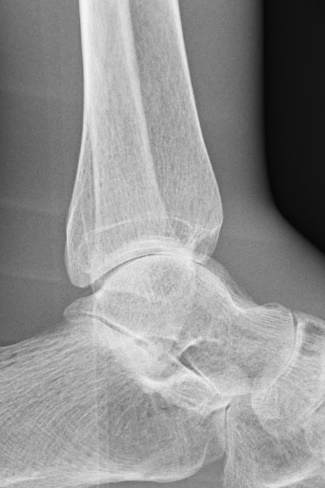 | 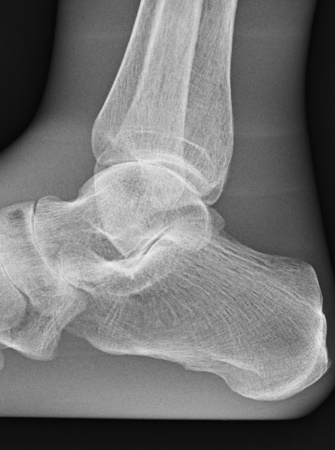 |
| --- | --- | --- |
| 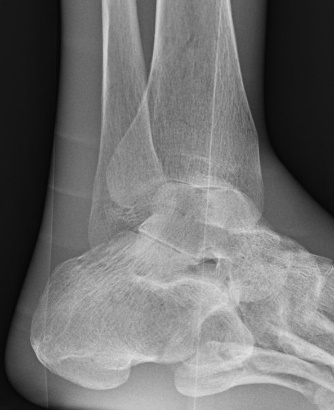 | 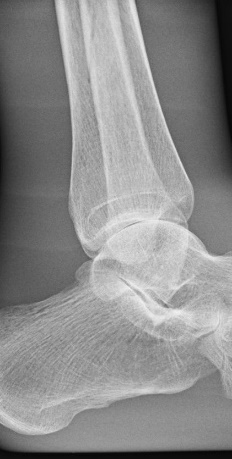 |  |

**Oblique hand**

Prompt: This is an oblique hand projection. In terms of radiographic positioning, please determine the errors, if any, in the radiograph? If there are errors, please determine the nature of the error(s) and provide instructions for a radiographer to improve the image.

| 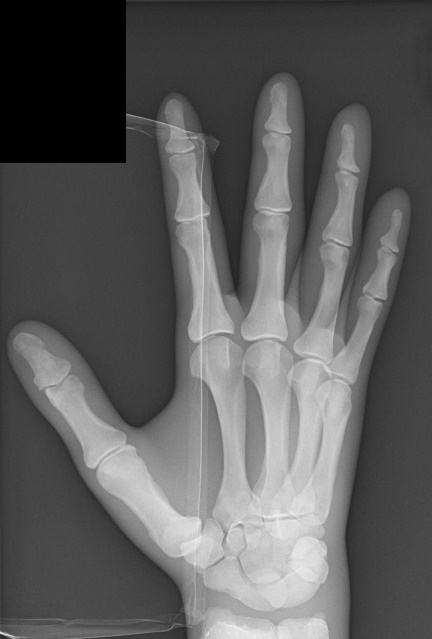 | 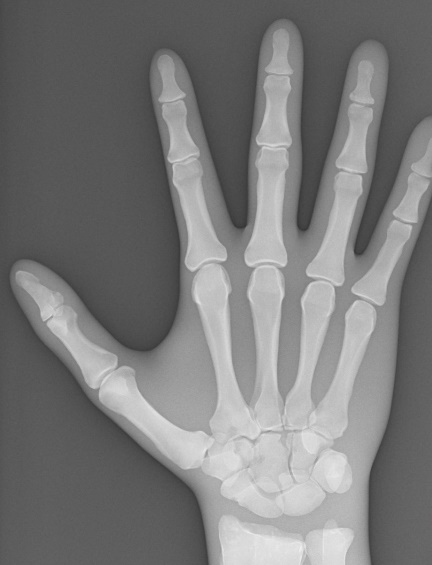 | 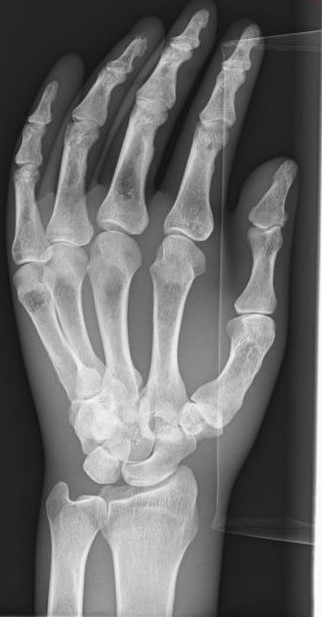 |
| --- | --- | --- |
| 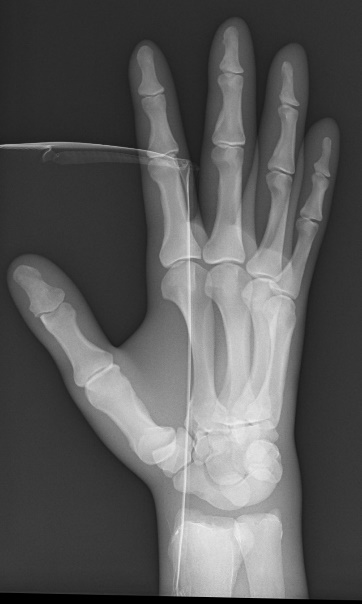 | 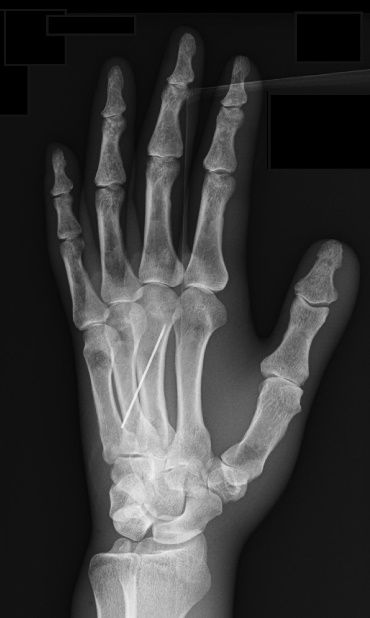 |  |

**Lateral knee**

Prompt: This is a horizontal ray lateral knee projection. In terms of radiographic positioning, please determine the errors, if any, in the radiograph? If there are errors, please determine the nature of the error(s) and provide instructions for a radiographer to improve the image.

| 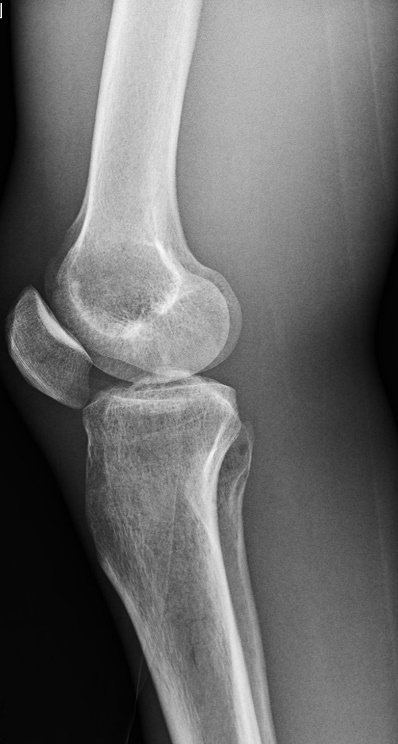 | 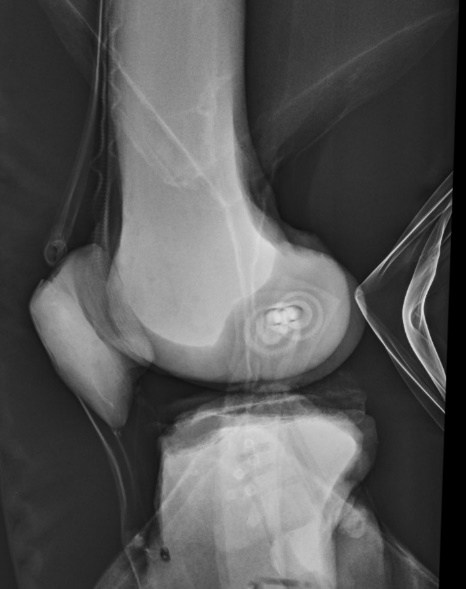 | 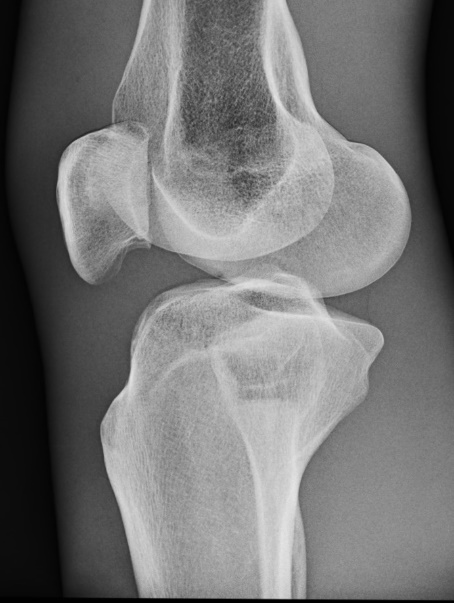 |
| --- | --- | --- |
| 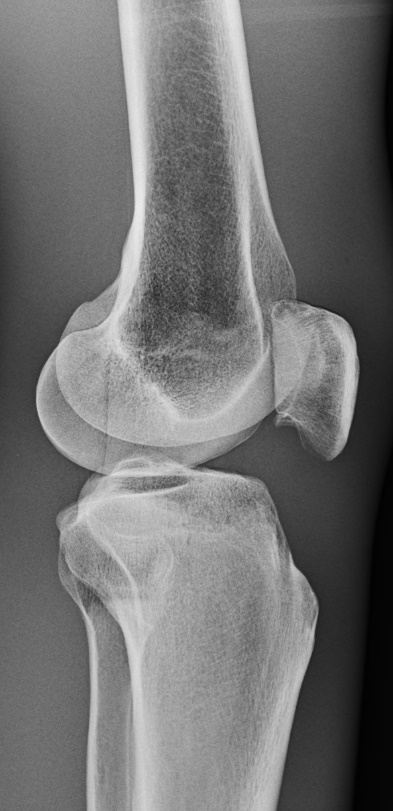 | 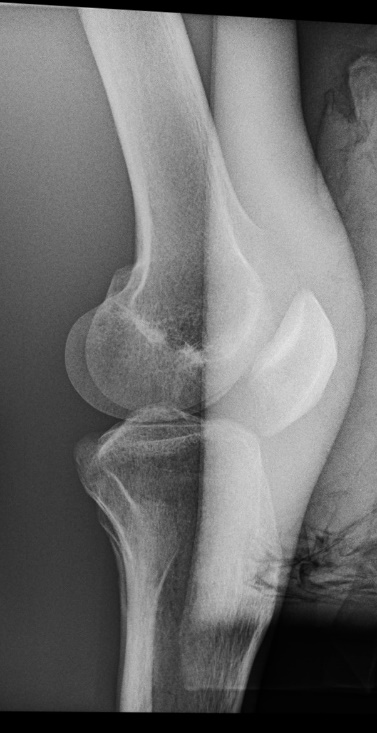 |  |

**Anteroposterior pelvis**

Prompt: This is an anteroposterior pelvis projection. In terms of radiographic positioning, please determine the errors, if any, in the radiograph? If there are errors, please determine the nature of the error(s) and provide instructions for a radiographer to improve the image.

| 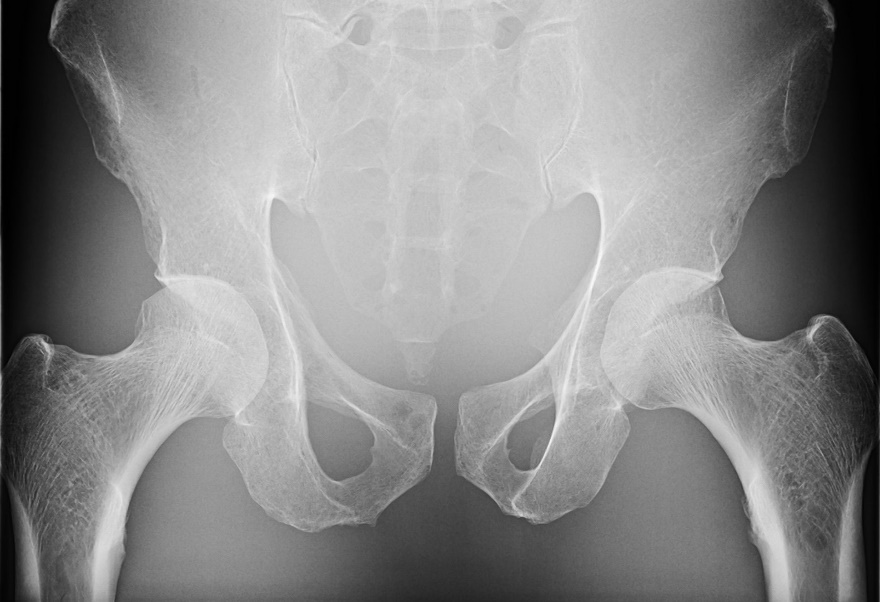 |
| --- |
| 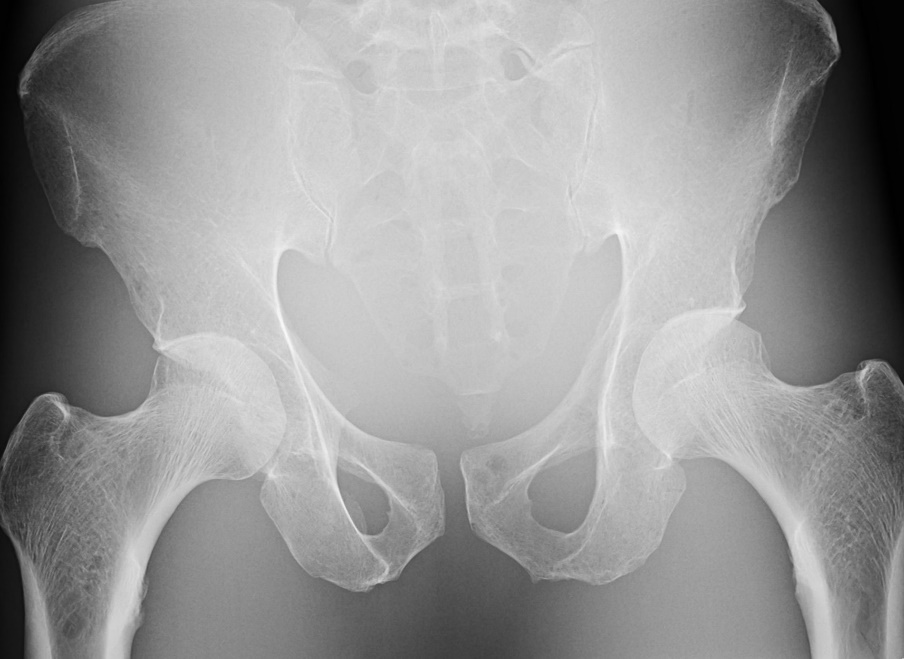 |
| 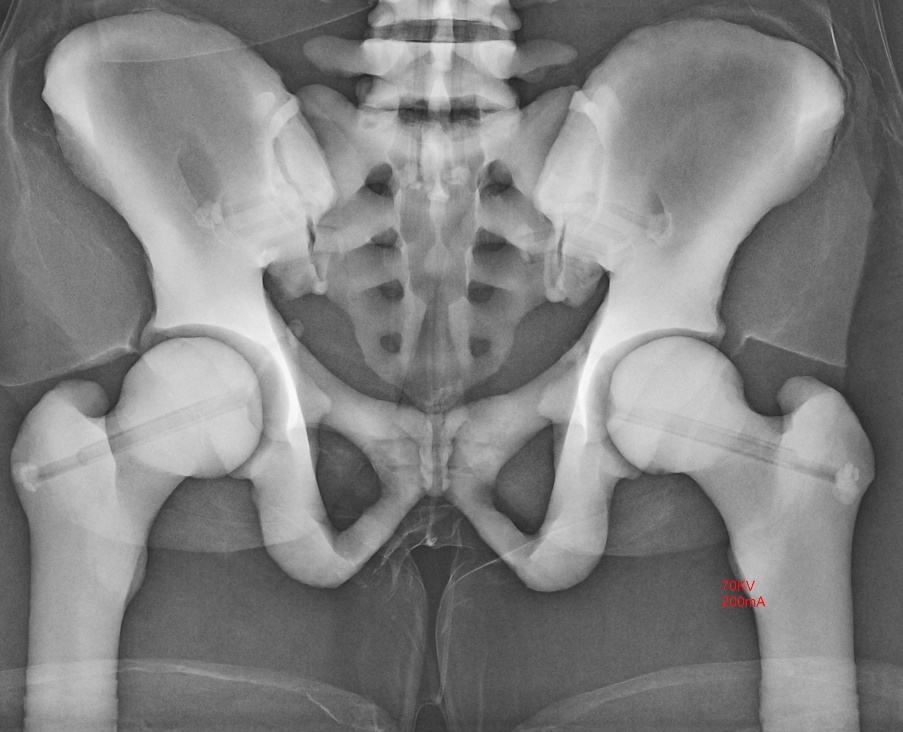 |
| 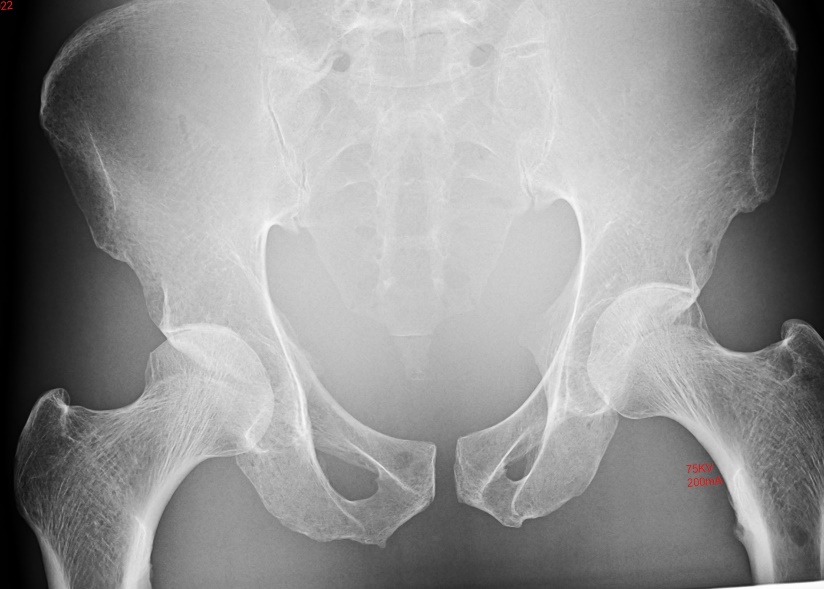 |
| 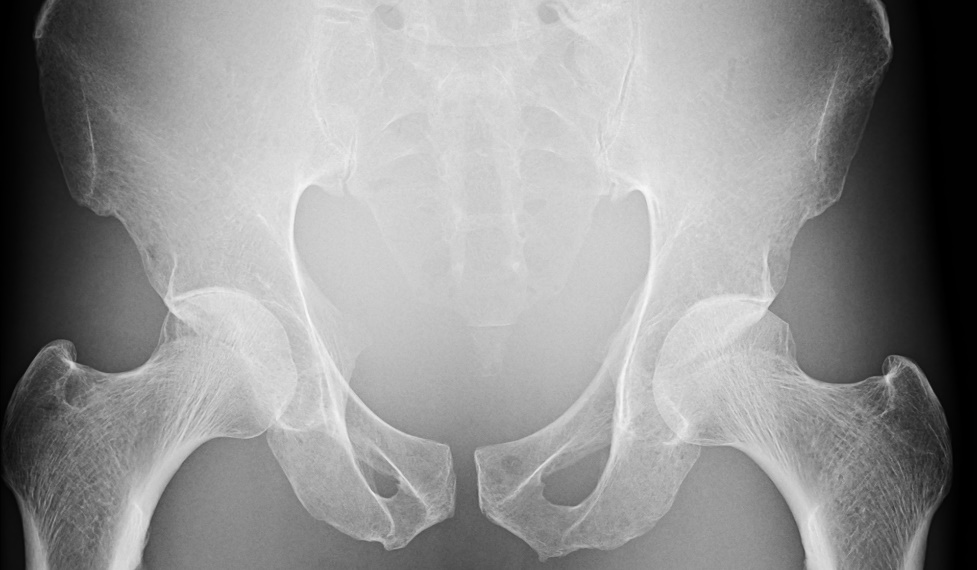 |

**Glenohumeral shoulder**

Prompt: This is a glenohumeral (Grashey) shoulder projection. In terms of radiographic positioning, please determine the errors, if any, in the radiograph? If there are errors, please determine the nature of the error(s) and provide instructions for a radiographer to improve the image.

| 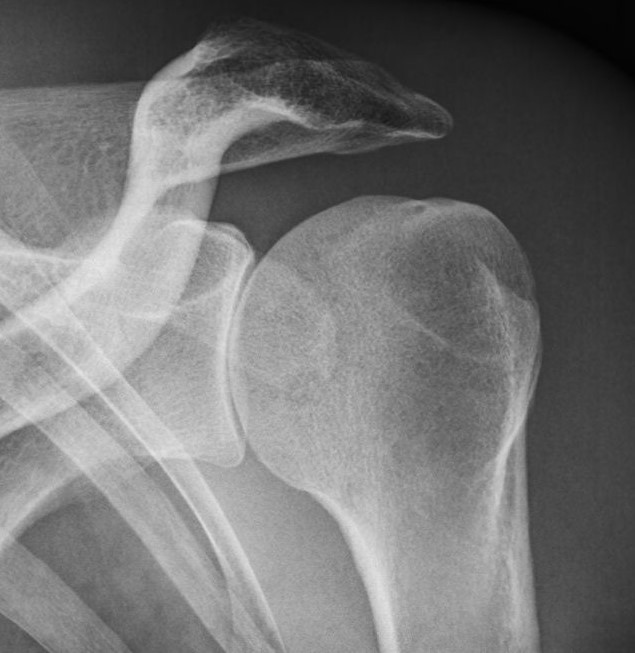 |
| --- |
| 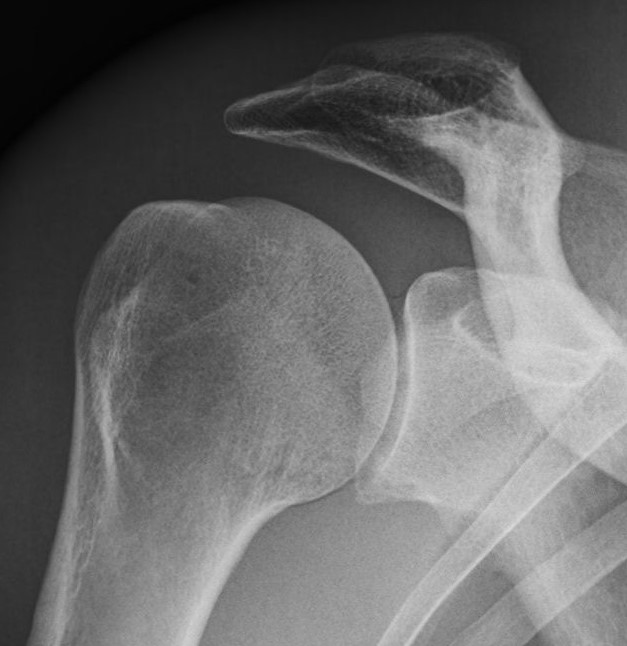 |
| 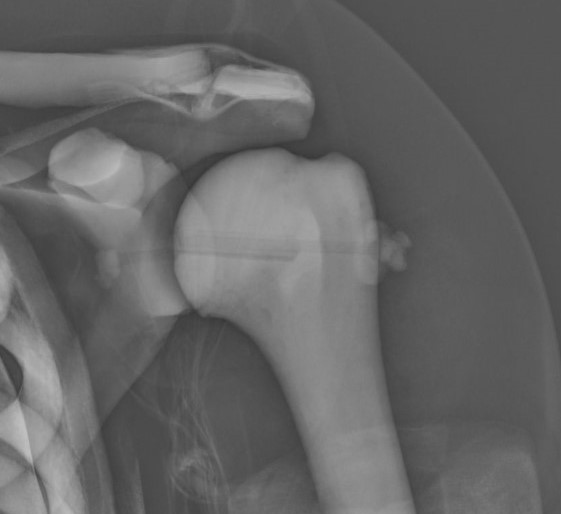 |
| 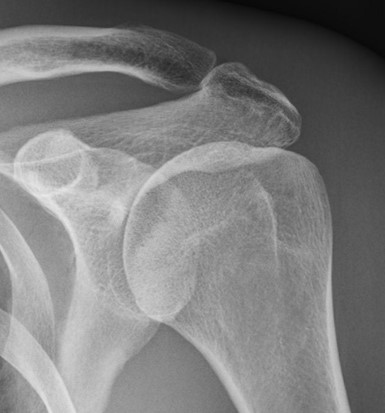 |
| 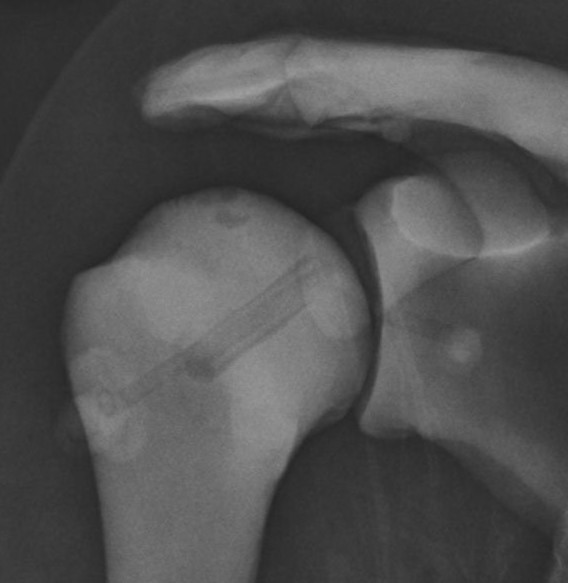 |
